# How is total antioxidant status in plasma of Patients with brucellosis?

**Published:** 2012

**Authors:** Mehdi Gholami, Mohammadreza Hasanjani Roushan, Soleiman Mahjoub, Ali Bijani

**Affiliations:** 1 Department of Biochemistry, Faculty of Medicine, Babol University of Medical Sciences, Babol, Iran.; 2 Infectious Diseases and Tropical Medicine Research Center, Babol University of Medical Sciences, Babol, Iran.; 3 Fatemeh Zahra Infertility and Reproductive Health Research Center, Babol University of Medical Sciences, Babol, Iran.; 4 None – Communicable Pediatrics Diseases Research Center, Babol University of Medical Sciences, Babol, Iran.

**Keywords:** Acute brucellosis, Free radical, Oxidative stress, Total antioxidant.

## Abstract

**Background:**

Brucella species can cause infection in a wide range of animals and human beings. Oxidative events against brucella infection are not well elucidated. It is possible that brucellosis may be related to increased free radical production and antioxidant depletion. Thus, the aim of this study was to determine the total antioxidant capacity (TAC) before and after treatment of patients with brucellosis.

**Methods:**

In the present study, a total of 48 patients with brucellosis, 23 males and 25 females, were detected through Wright ≥1/160 2ME ≥1/80, in association with compatible clinical findings. All patients were treated with standard regimens of therapy. Total antioxidant capacity (TAC) was determined with ferric reducing antioxidant power (FRAP) using spectrophotometric method before and after treatment.

**Results:**

TAC levels were significantly lower in pre-treatment than in post-treatment patients, 0.783±0.015 and 0.819±0.024 m mol/L respectively (p<0.01). There was not significant relation between plasma TAC levels and age or gender of the patients.

**Conclusion:**

TAC may be useful as an early marker of oxidative stress to monitor and optimize antioxidant therapy as an adjunct in the management of patients with brucellosis.

Brucellosis is a zoonotic infection with acute and chronic clinical complications. Each year more than 500000 new  cases of this disease with an uneven distribution is reported in the worldwide (1). Brucella species can cause infection in a wide range of animals and human beings. It remains a significant public health problem in the areas of the world where they are endemic in herbivorous animals (2). The majority of cases occur in the Mediterranean countries of Europe and Africa, the Middle East, India, Central Asia, Mexico, and the Central and South America (2). Iran is an endemic area for brucellosis (3). Brucella species are usually transmitted from infected animals to humans by ingestion of raw animal products and on rare occasion are transmitted by inhalation or by direct animal contact (4). The disease is characterized by undulant fever, arthritis, anxiety, sweating, malaise, anorexia, amnesia, delusions, hallucinations, delirium, phobias and irritability (5). The systemic complications include endocarditis, spondylitis, sacroiliitis, osteomyelitis, meningitis, and orchitis (3-6). Brucella a gram-negative, facultative, intracellular pathogen is able to survive and replicate into the host phagocytes despite the phagocytosis by macrophages and monocytes (7). Brucella pathogen can stimulate cerebral lipid peroxidation in the infection without causing significant inflammation (8). The escape mechanism of intracellular killing by polymorphonuclear is not well elucidated, but it has been reported that brucella through the lipid peroxidation and control of antioxidant systems in the host cell continue to survive (9, 10). It is possible that brucellosis may be related to increase free radical production and antioxidant depletion while oxidative stress may be implicated in the pathogenesis of brucellosis (10-11). 

Oxidative stress status in different diseases were investigated by Mahjoub et al. in the population of Babol, North of Iran (12-18). Since the precise oxidative stress data before and after treatment in acute brucellosis is not available, in the present study we evaluated the antioxidant status in patients with brucellosis before and after treatment. 

## Methods

 This prospective study was conducted in the Department of Clinical Biochemistry and Infectious Diseases & Tropical Medicine Research Center in Babol University of Medical Sciences from July 2009 to August 2011. Informed consents were obtained and the study protocol was approved by the Ethics Committee. A total of 48 patients with brucellosis, (23 males and 25 females), were diagnosed by the Wright test ≥1:160 2ME ≥1/80 in association with compatible clinical findings (e.g., fever, sweating, malaise, anorexia, headache, back pain). All the patients were treated with the standard regimens of therapy. Those who had spondylitis were treated with a longer duration therapy. A 5-ml sample of venous blood was obtained in heparinized tubes from each individual immediately upon the diagnosis of brucellosis before the treatment was initiated. Plasma was separated by centrifugation at 1500 x g for 10 min, then the samples were stored at –80°C until further analysis of TAC. The blood samples were obtained from the patients after brucellosis was identified after the end of treatment period. 

**Measurement of Total antioxidant capacity**

**FRAP assay: **Total antioxidant capacity was measured by FRAP assay according to Benzie and Strain method (19). Briefly, 1.5 mg FRAP ready to use reagent (acetate buffer, TPTZ reagent and Ferric chloride solution Ratio of 10:1:1, respectively) was added to all the test tubes and were incubated for 5 min at 37 °C. Then, 50 µl of samples or standards were added to the related tubes and were mixed gently, incubated for 10 min at 37°C. At low pH, when a ferric tripyridyltriazine (Fe^III^-TPTZ) complex is reduced to the ferrous (Fe^II^) form, an intense blue color with an absorption maximum at 593 nm developed and the color intensity was measured using spectrophotometer (Jenway 6505, UK). Absorbance values obtained from the standard curve (Iron sulfate as standard 125, 250, 500, 1000 µm) was converted to concentration.

**Statistical Analysis: **The collected data before and after treatment of the patients with brucellosis was evaluated with SPSS version 17. The data obtained from the study were compared using Chi square test, t-test, paired t-test and Pearson correlation. The differences were considered statistically significant at p<0.05.

## Results

 Forty-eight patients with brucellosis were investigated before and after treatment in the present study. The mean age of male and female patients was 45.74±16.41 years and 44.60±17.45, respectively. The male to female ratio of patients was 23/25 (p>0.05). The symptoms and signs of patients were fever (75%), sweating (78%), chills (25%) back pain (39%), arthralgia (32%), myalgia (20.8%), spondylitis (20.8%), loss of weight (20.8%), sacroiliitis (14.5%), less movement (12.5%), weakness (12.5%), nausea (8.3%), vomiting (6.25%) abdominal pain (6.25%), headache (6.25%), and splenomegaly (2.1%). Blood cultures were positive in (14.5%) of cases. More patients consumed raw milk or fresh cheese (83%) of cases. Close contact with animal and other risk factors consisted 17% of cases. We observed 6.25% of cases leukopenia, 4.16% leukosytosis and 20.8% anemia in those patients. CRP test was positive in 31 (64.6%) patients and the elevated levels of ESR were observed in 38 (79.1%) patients. RF test was positive only in 5 (10.4%) patients.

The FRAP standard curve that shown in Fig. I was obtained from Aborbance values of Iron sulfate as standard with serial concentrations (125, 250, 500, 1000 m). 

The values of plasma total antioxidant capacity in before after treatment of patients with acute brucellosis was shown in fig 2. TAC levels were significantly lower in pre-treatment than in post-treatment patients, 0.783±0.015 and 0.819±0.024 m mol/L respectively (p<0.01). 

**Figure 1:**
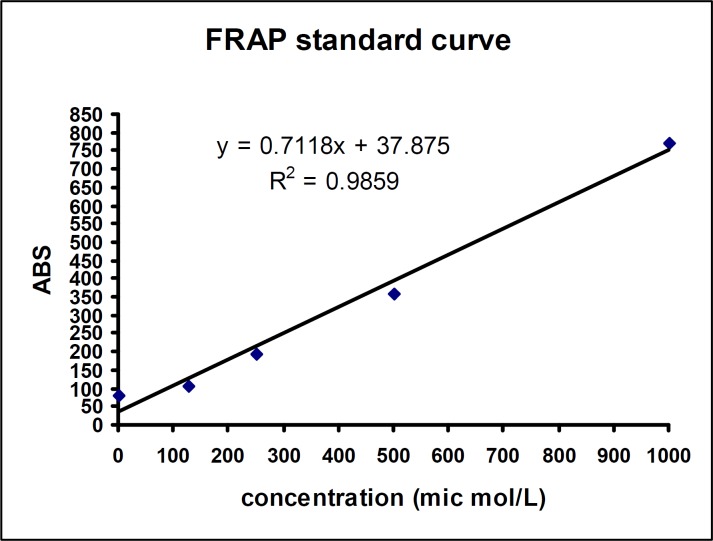
FRAP standard curve

**Figure 2:**
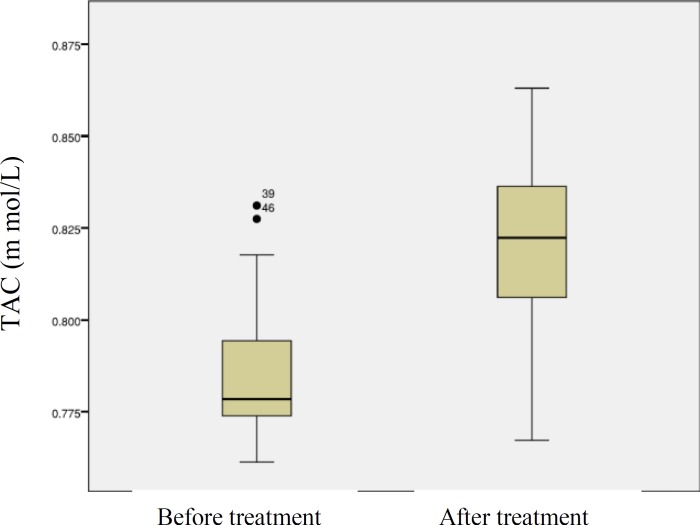
Box plot, Plasma TAC levels before and after treatment of patients with brucellosis (p<0.01

## Discussion

In order to investigate the antioxidant status in patients with brucellosis, we measured the total antioxidant capacity (TAC) before and after the defined treatment of the patients. There is no prior study investigating this biomarker of oxidative stress defense in patients with brucellosis before and after the defined treatment. Our study is one of the initial attempts to investigate the changes in TAC level in brucellosis and the effects of treatment on TAC status in the treated patients. 

In the present study, we found that TAC level was significantly decreased in the patients before treatment as compared to the treated patients indicating that the antioxidative system of these patients was impaired. It is known that various antioxidants in plasma have additive effects on oxidative status, and the cooperation of the antioxidants prevents adverse effects of free radicals (20). The measurement of individual antioxidant components may not accurately reflect the total antioxidant status and the measurement of TAC practically represents all of them and therefore can reflect the oxidative status of the organism (21-23). In the present study, we used a colorimetric measurement method developed by Benzie & Strain. The measurement of TAC levels with this method is simple, reliable and sensitive (19). A possible mechanism for the decreased level of TAC could be that the scavenger antioxidants were consumed by the increased free radical activity associated with Brucella infection. This opinion was supported by the fact that patients with brucellosis significantly increases in lipid peroxidation, and malondialdehyde. The oxidative stress is responsible for tissue damage in brucellosis patients (8, 10, 12). 

The reactive oxygen species that formed within cells can oxidize bio-molecules leading to cell death and tissue injury. Free radicals can attack almost any component of the cell, especially lipids, proteins, and nucleic acids (14-16). In some of the inflammatory and infectious diseases, ROS are not the initial cause of the disease but improve secondarily to the primary disorder and is involved in pathogenesis (13, 17, 22). Several clinical and experimental studies have tried to clarify the role of oxidative stress in the pathogenesis of many diseases (24, 25). There is a balance between reactive oxygen species (ROS) produced in human and animal bodies and antioxidant defense (26). 

In case of an increase in ROS production in aging, cancer and various infections or decrease in antioxidant defense, free oxygen radicals react with macromolecules that contain protein, lipid and DNA and cause oxidative stress as a tissue injury (14-16). Brucella evades host defences by inhibiting endosome fusion with lysosomes and finally may reach the endoplasmic reticulum. In fact, this bacteria may have an extreme preference for the intracellular environment despite their ability to live outside host cells. Thus, brucella inside the macrophage are protected not only from the immune system (antibodies, complement) but also from vitro active antibiotics that do not reach therapeutic concentrations into those intracellular compartments (20). 

Reactive oxygen species (ROS) are produced during many metabolic and physiological processes. Organisms have several enzymatic and non-enzymatic antioxidant systems that overwhelm harmful effects of these ROS. Under certain conditions, antioxidants mechanisms are impaired and/or ROS are increased and antioxidant mechanisms may become insufficient to prevent oxidative damage completely. Consequently, oxidative stress develops (21, 26). Free radicals produced by leukocytes and macrophages play a major role in killing intracellular pathogen. Brucella has the ability to survive and multiply against oxidative killing by several unclear mechanisms in the phagocytic cells of the host. Thereby, elevate free radicals by leukocytes due to lipid peroxidation and consequently inflammation and tissue damages (10, 20, 22). Also, we observed the elevation of CRP and ESR in these patients. These markers decreased in treated patients. It is known that CRP and ESR, recognized as traditional inflammation markers, increase in brucellosis cases and decrease to normal levels after treatment. It is reported in various studies that CRP especially is a useful marker of the activity of brucellosis and in monitoring the efficiency of the given treatment (27, 28). In a similar study like ours, Serefhannoglu et al. (10) reported an increase in MDA levels and total peroxide and decrease in TAC levels in brucellosis. Kilic et al. showed that MDA levels were high in acute brucellosis cases and dropped down to normal levels during recuperation after treatment (29). Also Karaagac et al. reported that total oxidant status (TOS) and OSI levels were significantly higher in patients with brucellosis before therapy as compared to the treated group. Whereas in contrast, total antioxidant capacity (TAC) levels were significantly higher in treated group (22). 

In conclusion, according to the data of the present study, decreased TAC levels showed that the patients with brucellosis were exposed to potent oxidative stress. There is a need for further studies for a more conclusive statement on this issue however, but our primary results may have importance with respect to the significance of oxidative stress in understanding the pathogenesis of acute brucellosis and therapeutic strategies. Also, TAC may be useful as an early marker of oxidative stress to monitor and optimize antioxidant therapy as an adjunct in the management of acute brucellosis patients.

## Notes

**Funding:** This project was financially supported by the Infectious, Diseases and Tropical Medicine Research Center of Babol University of Medical Sciences.

## References

[1] Skalsky K, Yahav D, Bishara J (2008). Treatment of human brucellosis: systematic review and meta- analysis of randomised controlled trials. Bmj.

[2] Young EJ (1995). An overview of human brucellosis. Clin Infect Dis.

[3] Hasanjani Roushan MR, Mohrez M, Smailnejad Gangi SM, Soleimani Amiri MJ, Hajiahmadi M (2004). Epidemiological features and clinical manifestations in 469 adult patients with brucellosis in Babol, Northern Iran. Epidemiol Infect.

[4] Al Dahouk S, Nöckler K, Hensel A (2005). Human brucellosis in a nonendemic country: a report from Germany, 2002 and 2003. Eur J Clin Microbiol Infect Dis.

[5] Parizadeh SMJ, Seyednozadi M, Erfanian MR, Azimi nezhad M (2009). A survey on Antibody levels among individuals at the risk of brucellosis in khorasan razavi province, Iran. Pakistan J Nutr.

[6] Balabanova-Stefanova M, Starova A, Arsovska-Bezhoska I (2010). Cutaneous Manifestations of Brucellosis. Macedonian J Med Sci.

[7] Pappas G, Papadimitriou P, Akritidis N, Christou L, Tsianos EV (2006). The new global map of human brucellosis. Lancet Infect Dis.

[8] Melek IM, Erdogan S, Celik S, Aslantas O, Duman T (2006). Evaluation of oxidative stress and inflammation in long term Brucella melitensis infection. Mol Cell Biochem.

[9] Lopez-Urrutia L, Alonso A, Nieto ML (2000). Lipopolysaccharides of Brucella abortus and Brucella melitensis Induce Nitric Oxide Synthesis in Rat Peritoneal Macrophages. Infect Immun.

[10] Serefhanoglu K, Taskin A, Turan H (2009). Evaluation of Oxidative Status in Patients with Brucellosis. Braz J Infect Dis.

[11] Kataria N, Kataria AK, Maan R, Gahlot AK (2010). Evaluation of oxidative stress in brucella infected cows. J Stress Physiol Biochem.

[12] Mahjoub S, Hasanjani Roushan MR, Gholami M (2011). Evaluation of oxidative stress before and after treatment of patients with acute brucellosis.

[13] Mahjoub S, Jalali F, Seyyedi A (2009). Status of Plasma Lipid Peroxidation in Patients with congestive Heart Failure. Eur J Cardiovasc Prev Rehabil.

[14] Mahjoub S, Tamadoni A, Gorji R (2009). Lipid and Protein Peroxidation in Patients with beta- Thalassemia Major and Intermedia. 3rd International Congress on Biochemistry and Molecular Biology (3rd ICBMB); 2009 Nov 16-19; Tehran, Iran. J Iranian Chem Society.

[15] Mahjoub S, Jalali F, Askari M (2008). Plasma Total Antioxidant Capacity in Patients with Congestive Heart Failure. 16th Congress of Iranian Heart Association in collaboration with American College of Cardiology; 2008 Nov 18-21; Tehran, Iran. Iranian Heart J.

[16] Mahjoub S, Tamadoni A, Nikoo M (2007). Iron overload and oxidative stress in beta-Thalassemia patients in north of Iran. 11th Asian Pacific Congress of Clinical Biochemistry; 2007 Oct 14-16; Beijing, China. Chinese Med J.

[17] Mahjoub S, Tamadoni A, Nikoo M, Moghadamnia AA (2007). Oral supplement of vitamin E and beta carotene reduce lipid & protein peroxidation of erythrocytes in beta-Thalassemia major patients. 9th Iranian Congress of Biochemistry and The Second International Congress of Biochemistry & Molecular Biology; 2007 Oct 29-Nov 1; Shiraz, Iran. Arch Iranian Med.

[18] Mahjoub S, Tamadoni A, Nikoo M, Moghadamnia AA (2007). The effects of beta carotene and vitamin E on erythrocytes lipid peroxidation in beta-thalassemia. J Res Med Sci.

[19] Benzie IF, Strain JJ (1996). The ferric reducing ability of plasma (FRAP) as a measure of 'antioxidant power': The FRAP assay. Anal Biochem.

[20] Lecaroz C, Blanco-Prieto MJ, Burrell MA, Gamazo C (2006). Intracellular killing of Brucella melitensis in human macrophages with microsphere-encapsulated gentamicin. J Antimicrob Chemother.

[21] Aruoma OI (1996). Characterization of drugs as antioxidant prophylactics. Free Radic Biol Med.

[22] Karaagac L, Koruk ST, Koruk I, Aksoy N (2011). Decreasing oxidative stress in response to treatment in patients with brucellosis. Int J Infect Dis.

[23] Cao G, Prior RL (1998). Comparison of different analytical methods for assessing total antioxidant capacity of human serum. Clin Chem.

[24] Aslan M, Kosecik M, Horoz M (2007). Assessment of paraoxonase and arylesterase activities in patients with iron deficiency anemia. Atherosclerosis.

[25] Erel O (2004). A novel automated direct measurement method for total antioxidant capacity using a new generation, more stable ABTS radical cation. Clin Biochem.

[26] Halliwell B (2006). Reactive species and antioxidants. Redox biology is a fundamental theme of aerobic life. Plant physiol.

[27] Dabdoob WA, Abdulla ZA (2000). A panel of eight tests in the serodiagnosis and immunological evaluation of acute brucellosis. East Mediterr Health J.

[28] Navarro JM, Mendoza J, Leiva J, Rodriguez-Contreras R, de la Rosa M (1990). C-reactive protein as a prognostic indicator in acute brucellosis. Diagn Microbiol Infect Dis.

[29] Kilic N, Ozden M, Kalkan A (2005). Lipid peroxidation levels in patients with acute brucellosis. Clin Exp Med.

